# Treatment with palbociclib and tislelizumab for CDKN2A-mutated and PD-L1-positive advanced intrahepatic cholangiocarcinoma: a case report and literature review

**DOI:** 10.3389/fonc.2024.1292319

**Published:** 2024-01-24

**Authors:** Yajun Liang, Liya Hu, Huanlei Wu, Tiejun Yin, Jun Zhang

**Affiliations:** ^1^ IV Ward of Pulmonary and Critial Care Medicine, Wuhan Pulmonary Hospotal, Wuhan, Hubei, China; ^2^ Department of Geriatrics, Tongji Hospital, Tongji Medical College, Huazhong University of Science and Technology, Wuhan, Hubei, China; ^3^ Department of Internal Medicine, The Fifth People’s Hospital of Jingzhou, Jingzhou, Hubei, China

**Keywords:** intrahepatic cholangiocarcinoma, targeted therapy, CDKN2A, immunotherapy, case report, literature review

## Abstract

**Background:**

Intrahepatic cholangiocarcinoma (ICC) is the second most common primary liver malignancy with a steadily increasing incidence worldwide. ICC has insidious onset, rapid progression, and poor prognosis. More multidisciplinary clinical studies are needed to continuously explore safer and more efficient diagnosis and treatment modes for ICC.

**Methods and results:**

A 66-year-old female patient with ICC rapidly developed systemic multiple metastases after surgery, and the first-line two-drug combination chemotherapy was not effective. Due to cyclin-dependent kinase inhibitor 2A mutation and programmed cell death-ligand 1-positive, a partial response and progression-free survival of 9.5 months were achieved after a second-line treatment with cyclin-dependent kinase 4/6 inhibitor (CDK4/6i) combined with immunotherapy. The patient developed thromboembolism 7 months after treatment and died due to disseminated intravascular coagulation.

**Conclusion:**

The combination of targeted and immune therapy has revealed a potentially effective regimen for the effective treatment of patients with ICC, which needs to be observed in larger clinical studies. The thromboembolism rates in real-world patients treated with CDK4/6 inhibitors are higher than those reported in clinical trials, and the application of prophylactic anticoagulation in this patient population may be questionable.

## Introduction

Intrahepatic cholangiocarcinoma (ICC) refers to tumors originating from the epithelium of the secondary bile duct and its branches and is a relatively rare malignant tumor in humans (1). The patients with cholangiocarcinoma in our country account for 55% of the cases worldwide. The peak incidence is from 55 to 75 years old, and the male-to-female ratio is about 2:3 (2). Histopathological types include adenocarcinoma, adenosquamous carcinoma, squamous cell carcinoma, mucinous carcinoma, and so on. Most of them are adenocarcinomas with varying levels of differentiation. Surgery is the preferred treatment to prolong the survival of ICC patients, but most patients have lost the opportunity to be operated on when they are found. The postoperative recurrence rate is as high as 70%, so the prognosis is very poor, and the 5-year overall survival rate is only 20% (3–5). Patients with advanced ICC have poor efficacy with a single treatment. The multi-disciplinary treatment (MDT)-based diagnosis and treatment mode combined with chemotherapy, targeted therapy, immunotherapy, and other treatment methods has become an important strategy to prolong the survival of ICC patients. Therefore, we report an ICC patient with CDKN2A mutation and PD-L1-positive expression who achieved partial response (PR) with a progression-free survival (PFS) of 9.5 months after a second-line therapy with cyclin-dependent kinase 4/6 (CDK4/6) inhibitor-targeted therapy (palbociclib) combined with immunotherapy (tislelizumab).

## Case presentation

**Figure 1:**
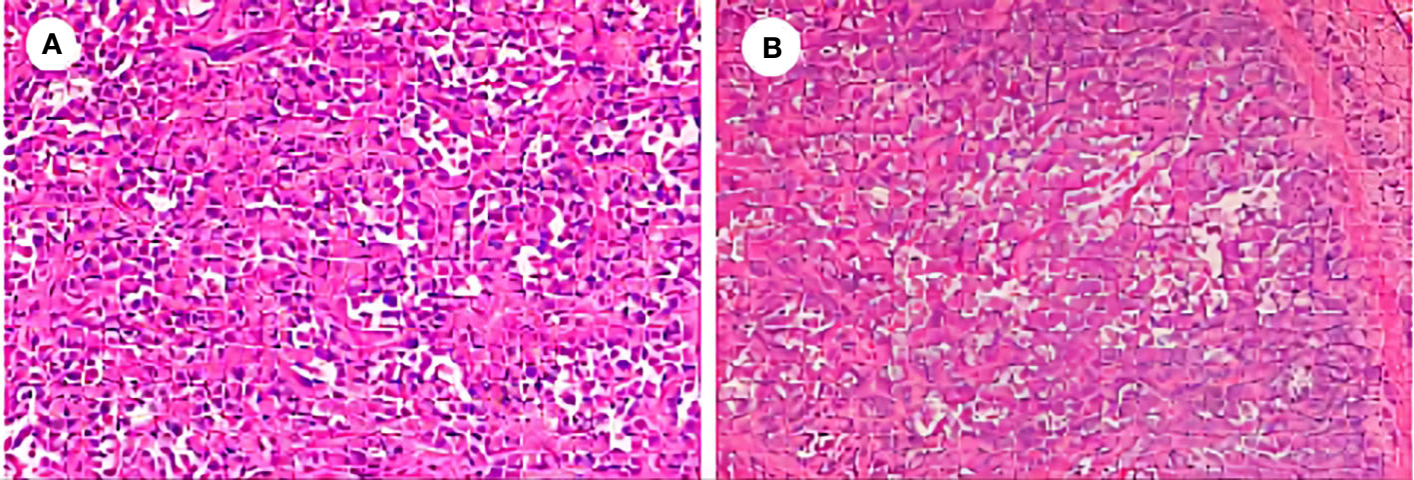
Postoperative pathological examination of the reported case. **(A)** Hematoxylin and eosin (H&E), ×200. **(B)** H&E, ×100.

A 66-year-old female patient went to the hospital on September 1, 2020 for a medical checkup, and an upper abdominal computed tomography (CT) scan revealed solid nodules in the right lobe of her liver. The carcino-embryonic antigen (CEA) level was 18 ng/mL (normal value: ≤5 ng/mL), and the carbohydrate antigen 19-9 (CA19-9) level was 2,865 U/mL (normal value: ≤34 U/mL). The positron emission tomography/computed tomography (PET/CT) result showed that the solid mass in the right lobe of the liver had a high metabolism (SUVmax: 12.3), indicating malignant neoplastic lesions. No obvious signs of malignant tumors were found in the other areas examined. Segmental hepatectomy was performed on September 9, 2020, and the postoperative pathological examination suggested that the liver lesion was poorly differentiated adenocarcinoma (CCA). Immunohistochemical (IHC) staining was positive for CK7 and CK19. However, arginase 1, GS, glypican-3, hepatocyte, CD31, CD34, and ERG were negative. The positive predictive value of Ki-67 was approximately 70% (**Figure 1**). The postoperative stage of the patient was pT1bN0M0 (stage IB). Because the tumor was in its early stage, the patient did not receive adjuvant therapy after surgery.

The patient developed abdominal pain and distension on October 28, 2020, and systemic imaging revealed rapid tumor progression. The chest CT examination showed scattered solid and subsolid density nodules in both lungs, suggesting intrapulmonary metastasis. The mediastinal lymph nodes were increased and enlarged, and metastasis was considered according to the history. The interlobular septa of both lungs were thickened, and lymphatic invasion was suspected. Liver magnetic resonance imaging (MRI) suggested multiple metastatic tumors in the liver as well as metastatic nodules in the liver capsule and peritoneum. A bone scan revealed multiple bone metastases throughout the body, including the Th5, Th6, and Th10 vertebrae, the left ilium, sacrum, bilateral acetabulum, right femoral head, left femoral neck, and bilateral ischium. The CEA and CA19-9 values were 15.91 ng/mL and 3,074 U/mL, respectively. The patient started the first cycle of chemotherapy with AG regimen (abraxane: 130 mg/m^2^ d1,8; gemcitabine: 1,000 mg/m^2^ d1,8 q3w) on November 10 and developed grade IV neutropenia and fever which recovered after treatment with colony-stimulating factor.

**Figure 2:**
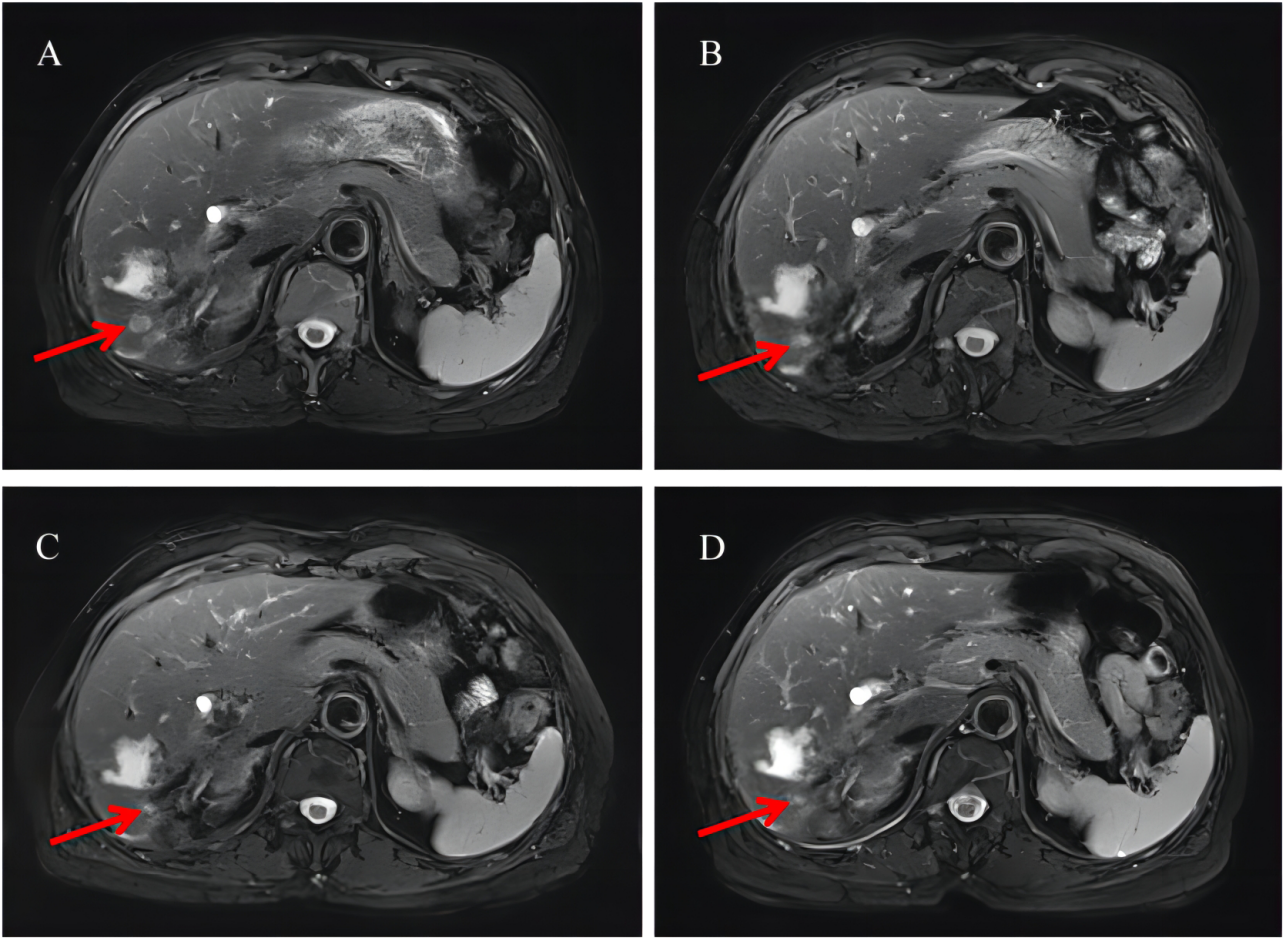
Radiological examination of the patient. **(A)** Liver MRI after one cycle of chemotherapy. **(B)** Liver MRI after combination therapy for 2 months. **(C)** Liver MRI after combination therapy for 4 months. **(D)** Liver MRI after combination therapy for 8 months. MRI, magnetic resonance imaging. The red arrow in the figure points to the metastatic tumor in the right posterior lobe of the liver.

On December 4, 2020, the patient developed limited tongue movement and numbness in the right jaw and facial area. The physical examination showed mild atrophy of the right tongue and poor movement, dull sensation in the right lower lip, and shallow sensation in the right peripheral trigeminal nerve and mandibular branch. The CEA and CA19-9 values were 14.98 ng/mL and 2,914 U/mL, respectively. She was diagnosed with right lingual paralysis (damage to the hypoglossal nerve) and numbness in her lower lip on the right side (damage to the mandibular branch of the trigeminal nerve). The patient was treated with vitamin B1 and B12 for two weeks, but there was no significant improvement in the numbness and discomfort. On December 21, the brain MRI re-examination revealed bilateral frontal lobe abnormal signal with enhancement. Abnormal bone signals were found around the sublingual nerve foramina of the right occipital condyle with irregular enhancement, unclear boundary, and limited diffusion. The size of the lesion was 25 × 9 mm, involving the sublingual nerve, and the potential for metastasis was taken into account. Based on the patient’s symptoms, signs, and imaging findings, the progression of the disease was assessed. Chemotherapy was ineffective, and the patient had a poor tolerance to previous treatments, so chemotherapy was discontinued. Surgical specimens were analyzed using next-generation sequencing techniques to identify possible molecular-targeted variants. The genomic profiling results were as follows: gene variation: CDKN2A p.W15, mutation frequency: 20.4%; and gene variation: BRIP1 p.A60Sfs, mutation frequency: 5.1%. In addition, the PD-L1 expression level of patient tumor tissue was detected, and the IHC staining results of tissue sections revealed a tumor proportion score (TPS) of 25% and a combined positive score (CPS) of 40% for PD-L1. The routine blood counts, coagulation function, liver function, and kidney function were normal. Based on the abovementioned results, the patient began to receive targeted therapy (palbociclib administered orally each day at a dose of 100 mg per day) and immunotherapy (tislelizumab administered 200 mg as an intravenous infusion over 30 min every 3 weeks). Brain MRI, liver MRI, and chest CT were reviewed every 2 months to evaluate changes in target lesions. RECIST criteria were used for the response evaluation. The patient provided consent, and the Institutional Review Board of Tongji Hospital approved this case study. On February 5, 2021, the patient reported a significantly reduced range of numbness in the right facial and mandibular region, and the tongue extension movement was free. A reexamination of the brain MRI showed no abnormal enhancement foci in the bilateral frontal lobes and no apparent diffusion-limited signal foci. The liver MRI suggested that the metastatic tumor in the right posterior lobe of the liver had significantly decreased in size compared to before. However, the presence of multiple nodules in the liver capsule and peritoneum was not clearly visible on the scan. The chest CT revealed that bilateral pulmonary nodules and enlarged lymph nodes were not clearly identified. The CEA and CA19-9 values were 3.25 ng/mL and 48.71 U/mL, significantly lower than before. The CEA level dropped to the normal range. The comprehensive assessment of the disease was PR, and immunotherapy and molecular targeted therapy were continued as planned. The re-examination in April and June 2021 showed that the brain enhancement focus was still gradually reduced, and the signal range of multiple bone abnormalities in the lumbar spine, sacral vertebra, and pelvis was slightly reduced than before (**Figure 2**).

**Figure 3:**
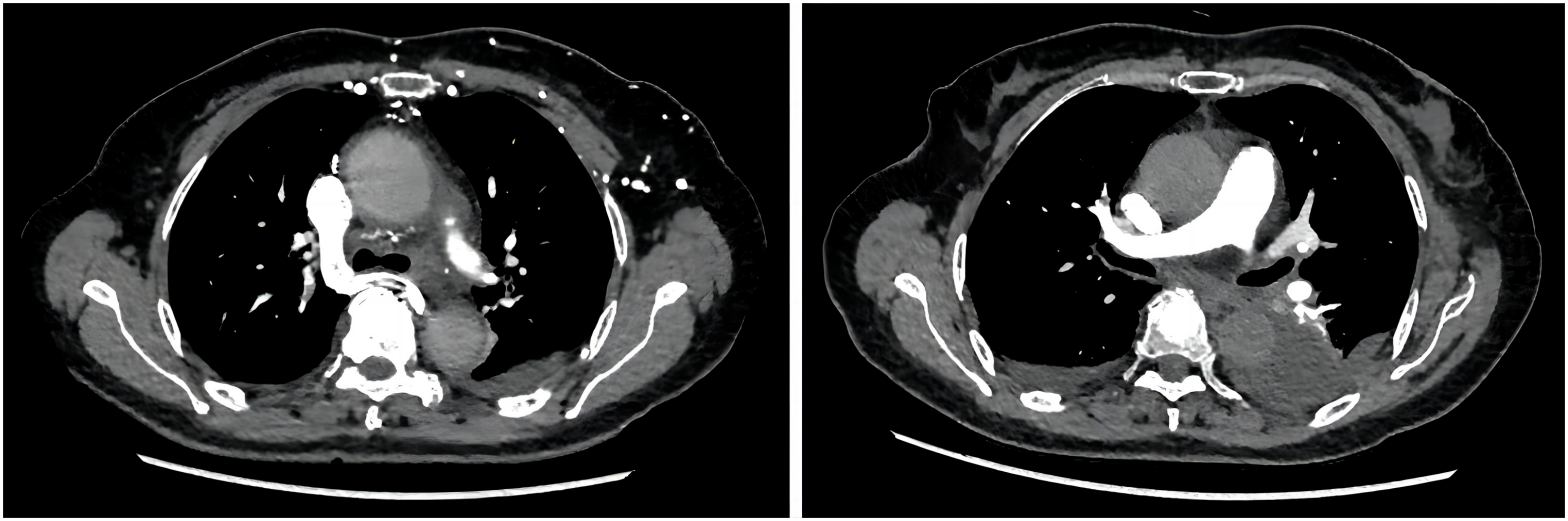
The computed tomography angiography examination revealed plaque-filled defects in the branches of the pulmonary artery.

**Figure 4:**
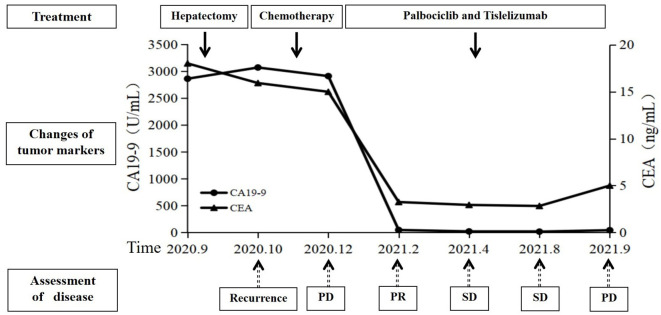
Summary of the treatment timeline of this case, including the levels of carcinoembryonic antigen (CEA) and carbohydrate antigen 199 (CA19-9). SD, stable disease; PD, progressive disease; PR, partial response.

By July 2, the patient complained of mild dysphagia, and a physical examination revealed swelling in the neck. The D-D dimer in blood coagulation function test was 11.52 ug/mL FEU, higher than the upper limit of normal value (0.5 ug/mL FEU) significantly. The patient’s routine blood counts, liver function, and kidney function were normal. A color ultrasound examination of the jugular vein indicated thrombus formation in the left internal jugular vein, subclavian vein, and innominate vein. The computed tomography angiography examination revealed small plaque-filled defects in grade 3–4 branches of the pulmonary artery in the upper and lower lobes of both lungs. Pulmonary embolism was diagnosed, and heparin anticoagulant therapy was given (**Figure 3**). The lesions were evaluated as stable disease by brain and liver MRI and chest CT in August 2021. The CEA and CA19-9 levels were normal. By September 18, the blood routine showed a platelet count of 60 * 10^9^/L↓, serum alanine aminotransferase was 821 U/L↑, total bilirubin was 30.0 umol/L↑, and fibrinogen was 0.63 g/L↓↓↓. The brain MRI revealed a strengthened nodule in the right occipital lobe with peripheral edema, which was considered a new metastatic tumor. The liver MRI showed a significant increase in retroperitoneal lymph nodes compared to the anterior. The pelvic MRI suggested increased retroperitoneal and bilateral para-iliac lymph nodes. The CEA level was normal, and the CA19-9 value was 44.19 U/mL. The disease was evaluated as progressive disease (PD). Considering that the patient had grade III bone marrow suppression, grade IV liver dysfunction, and efficacy was assessed as PD, the immune and targeted therapy, respectively, were discontinued, and symptomatic and supportive treatments such as liver protection were given. By September 24, the patient began to show symptoms of gastrointestinal, urinary, and vaginal bleeding successively and was diagnosed with disseminated intravascular coagulation (DIC). The patient’s condition gradually deteriorated, and she passed away by October 26 (**Figure 4**).

## Discussion

Intrahepatic cholangiocarcinoma originates from intrahepatic bile duct epithelial cells. The incidence of ICC is only lower than that of hepatocellular carcinoma, accounting for 10% to 15% of primary liver cancer, and the incidence is significantly increasing worldwide (6). The onset of ICC is insidious, and there are often no special clinical symptoms in the early stage. ICC is often found incidentally in imaging examination as an isolated intrahepatic mass, and the disease progresses rapidly. The most common type was adenocarcinoma, and immunohistochemical staining of tumor cells was positive for EMA, CK7, and CK19 (7). CA19-9 and CEA are the most commonly used serum markers for the diagnosis of ICC. Although their specificity is not strong, they are still valuable for diagnosis and treatment. Retrospective study results showed that postoperative dynamic monitoring of CA19-9 had a reference value for evaluating tumor residual or recurrence and predicting the prognosis of patients (8).

Patients with advanced ICC are mainly treated with systemic therapy, and single therapy is not effective. Two-drug combined chemotherapy is the main first-line standard treatment (9). The results of several clinical trials showed that chemotherapy combined with immunotherapy is effective for patients with biliary tract tumors. Camrelizumab plus GEMOX demonstrated a promising antitumor activity and an acceptable safety profile as a first-line treatment in advanced biliary tract cancer patients (10). ICC is associated with a variety of genetic variants, including mutations or fusions, and the corresponding targeted therapy regimens for these variants have been demonstrated to be effective in patients with ICC in phase II and III trials. These targets include IDH, FGFR, NTRK, HER2, and others (11–14). In clinical practice, more attention should be given to genetic testing and molecular targeted testing. It is recommended to implement MDT in the entire treatment process, ensuring individualized and reasonable selection for patients. This includes the combined application of chemotherapy, targeted therapy, immunotherapy, and other treatment methods.

Tislelizumab is a PD-1 monoclonal antibody that was originally developed in China. Based on its unique molecular structure, it has shown excellent efficacy and good safety. By modifying the Fc segment, the binding ability of FcγR was removed, antibody-dependent cell-mediated phagocytosis (ADCP effect) was eliminated, the anti-tumor efficacy was avoided due to the reduction of the number of T cells, and the adverse reactions caused by macrophages were avoided. Because its Fab segment has a larger surface area containing the PD-1 binding site, it exhibits a higher affinity for PD-1 and effectively inhibits the binding of PD-1 and PD-L1. In addition, the half-life of tislelizumab was longer than that of similar drugs in the highest range. Furthermore, the IC50 and EC50 values of tislelizumab were in the lowest range among similar drugs, indicating its strong anti-tumor activity. Clinical studies have shown that tislelizumab combined with first-line chemotherapy in the treatment of potentially resectable locally advanced biliary tract malignant tumors has an objective response rate of up to 56%, a disease control rate of 92%, and a R0 resection rate of 52%, with controllable safety (15). In another study of tislelizumab in the treatment of advanced biliary tract cancers, the conversion rate was 23.1% and the 6-month overall survival rate was 90.9% (95% CI, 50.8%–98.7%) (16). Its potent tumor reduction, safety, and effectiveness are surprising.

The CDKN2A gene is a frequently mutated gene in malignant tumors, commonly experiencing deletions and mutations in various types of tumors, including breast cancer and lung cancer. When the CDKN2A gene is deleted or mutated, it can result in the abnormal activation of cyclin-dependent kinases. Therefore, cyclin-dependent kinase inhibitors like palbociclib may hold promise as a potential treatment option. The previous literature reported that the clinical trial of palbociclib monotherapy in patients with cholangiocarcinoma showed no response and poor efficacy, leading to the closure of the clinical trial (17). However, a successful case of PR has also been reported (18). Palbociclib can activate T cells, promote the immune system’s ability to kill tumor cells, and enhance the effectiveness of immunotherapy (19). It has been reported that the CDK4/6 pathway is feasible as a potential target for treatment of patients with bladder cancer, especially when used in combination with immunotherapy (20). A patient with advanced lung squamous cell carcinoma had the CDKN2A mutation and a tumor proportion score of 80%. The patient responded well to CDK4/6 inhibitor abemaciclib therapy after experiencing disease progression with multiple chemotherapies and immunotherapy. A subsequent rechallenge with combined immunotherapy using Opdivo and ipilimumab resulted in a durable partial response (21).

The main adverse effect of palbociclib is neutropenia, which can be effectively managed by reducing the dosage of palbociclib. Palbociclib-induced liver function abnormalities mostly manifest as asymptomatic elevation of aminotransferase levels and rarely result in grade 3 elevation of aminotransferase. Drugs used in clinical liver protection can be used to treat CDK4/6 inhibitor-induced liver function abnormalities. Venous thromboembolism (VTE) events include deep vein thrombosis, subclavian vein thrombosis, and pulmonary embolism. Thrombosis is the second leading cause of death in cancer patients. During treatment, it is necessary to pay attention to and prevent the occurrence of VTE, monitor the symptoms and indications of deep vein thrombosis and pulmonary embolism in patients, and timely consult the relevant departments and take drug treatment if there is any abnormality. If VTE is not life-threatening, anti-tumor therapy can be continued in combination with antithrombotic therapy. A retrospective cohort study included 424 patients, of whom palbociclib was the most commonly used CDK4/6 inhibitor (n = 390, 91.8%). Venous thromboembolism occurred during CDK4/6 inhibitor use in 38 of 390 patients, with a rate of 6.3% at the first year. DVT, PE, and VVT accounted for 52.6%, 18.5%, and 15.8%, respectively. In real-world studies, the rates of VTE in patients with metastatic breast cancer treated with CDK4/6 inhibitors were found to be two to five times higher than what was reported in registered trials (22). The other study included 266 patients, with 89% of them in the palbociclib group. Out of these, 26 women (9.8%) experienced thrombotic events. Of these, 72% were venous thromboembolism. The incidence of thrombosis after 1 year was 10.4% overall and 10.9% in the palbociclib group, which was higher than that reported in clinical trials. Arterial thrombosis accounted for more than one-third of events, with the highest rate in the palbociclib group (23).

In this case, a liver-space-occupying lesion was accidentally found during routine physical examination, and surgery was performed in time. The postoperative pathological examination confirmed the diagnosis of ICC. At 1 month after surgery, the disease recurred rapidly and multiple metastatic lesions occurred throughout the body. The patient had a poor tolerance to the first-line treatment, which consisted of a combination of two drugs along with AG chemotherapy. Additionally, the treatment showed poor efficacy. There were still multiple new metastatic lesions, indicating that the disease was extremely malignant and progressed rapidly. According to the NGS test results, the patient switched to immunotherapy (tislelizumab) in combination with targeted therapy (palbociclib), which resulted in a rapid response. The patient’s discomfort symptoms were gradually relieved, and the blood levels of CA19-9 and CEA gradually decreased to normal. The imaging evaluation showed a PR 2 months later, and the PFS was as long as 9.5 months. Unfortunately, the patient developed thrombosis in the left internal jugular vein, subclavian vein, and innominate vein and pulmonary embolism 7 months after treatment. Despite receiving standard anticoagulant treatment, the effect was still not satisfactory. Later, there was a grade IV liver function abnormality and uncorrectable DIC, which occurred successively, and unfortunately, the patient was deceased in the end.

## Concluding remarks

ICC is a highly aggressive tumor of the digestive system, with a low rate of early diagnosis, rapid disease progression, and an improved prognosis. MDT is an ideal treatment mode to further improve the efficacy of ICC, and targeted therapy for ICC gene mutation combined with immunotherapy has become an alternative second-line treatment. This is the first report of palbociclib combined with tislelizumab for advanced metastatic intrahepatic cholangiocarcinoma. The identification of targetable molecular signatures and the combination of targeted therapy and immunotherapy reveal a potentially option for the effective treatment of patients with intrahepatic cholangiocarcinoma and should be considered after the failure of standard chemotherapy, but larger clinical studies are needed to make further observations. The thromboembolism rates in real-world patients treated with CDK4/6 inhibitors are higher than those reported in clinical trials, and the role of prophylactic anticoagulation in this patient population may be questionable.

## Data availability statement

The original contributions presented in the study are included in the article/supplementary material. Further inquiries can be directed to the corresponding author.

## Ethics statement

The studies involving humans were approved by the Institutional Research Ethics Committee of Tongji Hospital. The studies were conducted in accordance with the local legislation and institutional requirements. Written informed consent for participation in this study was provided by the participants’ legal guardians/next of kin. Written informed consent was obtained from the minor(s)’ legal guardian/next of kin for the publication of any potentially identifiable images or data included in this article.

## Author contributions

YL: Writing – original draft. LH: Writing – original draft. HW: Writing – original draft. TY: Writing – review & editing. JZ: Writing – review & editing.
